# Microbial Contamination on High-Touch Surfaces in Outpatient Clinics: Identification of Bacterial Strains from Areas of Patient and Medical Staff Occupancy

**DOI:** 10.3390/microorganisms13030698

**Published:** 2025-03-20

**Authors:** Karolina Prasek, Iwona Kiersnowska, Jadwiga Wójkowska-Mach, Anna Różańska, Dorota Romaniszyn, Ewelina Foryciarz, Lucyna Barbara Kwiećkowska, Edyta Krzych-Fałta

**Affiliations:** 1 Department of Nursing Propedeutics, Faculty of Health Sciences, Medical University of Warsaw, 01-445 Warsaw, Poland; karolina.prasek@wum.edu.pl (K.P.); lucyna.kwieckowska@wum.edu.pl (L.B.K.); edyta.krzych-falta@wum.edu.pl (E.K.-F.); 2 Department of Microbiology, Faculty of Medicine, Jagiellonian University Medical College, 31-121 Krakow, Poland; jadwiga.wojkowska-mach@uj.edu.pl (J.W.-M.); d.romaniszyn@uj.edu.pl (D.R.); 3 Medicover, 00-807 Warsaw, Poland; ewelina.foryciarz@medicover.pl

**Keywords:** outpatient care, hygiene, environmental microorganisms

## Abstract

Clinics and hospitals inherently increase the risk of adverse events, including hospital-acquired infections (HAIs) transmitted between healthcare personnel and patients. This study aimed to identify bacterial strains present on frequently touched surfaces in outpatient clinics used by patients as well as medical and non-medical personnel. This study was conducted in four outpatient care centers located in two major cities in Poland. A total of 85 samples were collected from frequently touched surfaces, including 53 samples from areas accessed by patients and 32 samples from surfaces used by medical staff. A statistically significant increase in moderate-to-heavy growth was observed in samples containing microbiota compared to those containing other microorganisms (*p* = 0.003). Similarly, a higher prevalence of spore-forming bacteria was noted compared to non-spore-forming bacteria (*p* = 0.001). A significant difference was also observed between samples with no or scant growth versus those with moderate-to-heavy growth in both the microbiota and other microorganism groups (*p* = 0.003), as well as between the spore-forming and non-spore-forming groups (*p* = 0.001). The findings of this study prompted revisions in cleaning procedures. The frequency of training for medical staff was increased, and systematic quality control of the cleaning company’s performance was implemented.

## 1. Introduction

Healthcare facilities, including outpatient clinics and hospitals, are crucial in providing medical care to patients with various diseases and conditions. However, during treatment in these institutions, patients are at risk of infections caused by different microorganisms, including those resistant to antibiotics. Hospital-acquired infections (HAIs) represent a significant public health concern, leading to prolonged hospital stays, increased treatment costs, and diminished quality of life for patients [1,2].

Many believe the risk of HAIs is confined primarily to the hospital environment. However, recent studies suggest that hospital-associated microorganisms can be transmitted from hospital settings to outpatient environments, such as clinics, dental offices, and rehabilitation centers. This transmission of microorganisms from hospital to outpatient settings is not only an epidemiological issue but also a clinical concern, as it can result in new infections and complications in patients who were not previously hospitalized [3].

Clinics and hospitals inherently increase the risk of adverse events such as HAIs transmitted from healthcare personnel to patients and vice versa [4]. Patients utilizing medical services and healthcare personnel providing those services may transmit microorganisms contributing to the development of both hospital-acquired and community-acquired infections, as well as microorganisms that are part of the human microbiome [5].

The microbiome consists of various bacteria, archaea, fungi, protozoa, and viruses. The composition of the microbiota is dynamic and constantly changing in response to external factors, such as nutritional status, environmental factors, lifestyle, and health, as well as diseases and medications such as antibiotics. The microbiota colonizes the gut, oral cavity, nostrils, vagina, and skin. Hospital-associated bacterial flora and microorganisms constituting the human microbiome naturally colonize contact surfaces in areas where patients and healthcare personnel are present. This is a natural and persistent phenomenon in places frequented by patients and personnel, including outpatient facilities [6,7].

Microorganisms transmitted by patients and staff can produce spores. Spore-forming bacteria are able to survive in extreme environmental conditions and can live in the environment for a very long time. Bacterial spores are resistant to disinfectants and are also able to survive life processes and spread through the food chain, causing severe infections of the digestive system [8].

The effectiveness of eradication is influenced by the number of microorganisms, the resistance of the microorganisms to disinfectants, and the strength and concentration of the disinfectant. On the other hand, following the procedures provided by the manufacturer of the preparation guarantees an effective disinfection process. At the same time, effective disinfection should reduce or eliminate the risk of infection [5]. Hower, it is known that traditional disinfection is susceptible to performance error, and this is one of the reasons, together with unsatisfactory compliance of healthcare workers with hand hygiene recommendations [9], why there are pathogenic bacteria quite commonly present in the healthcare environment [10,11,12]. The target of studies is usually the hospital environment due to the specificity of hospital treatment and antimicrobial resistance, which constitutes a very important issue in Poland where we face rates placed in the upper values of data on antimicrobial resistance in Europe [13,14] and *Clostridioides difficile* infections [15]. However, the outpatient setting should not be omitted in the description and diagnosis of the state of infection control and prevention. In Poland for example, these are outpatient settings which are characterized by higher than European average levels of antibiotic consumption comparing to hospitals [16]. However, there are many areas of unknown status of implementation of infection control and prevention in outpatient settings: among others, compliance with hand hygiene, environmental contamination and disinfection effectiveness.

This study aimed to identify bacterial strains present on frequently touched surfaces in outpatient clinics used by both patients and medical and non-medical personnel, as a pilot for potential further studies and infection control policy.

## 2. Materials and Methods

### 2.1. Statistical Analysis

Qualitative variables were compared using the chi-square test with a correction for low frequencies, while quantitative variables were analyzed using the Mann–Whitney U test. The statistical power was set at 0.8, and the significance level was established at 0.05. Calculations were performed using Statistica 13.3 software.

### 2.2. Study Design

This study was conducted in four outpatient care centers located in two major cities in Poland. Samples were collected and analyzed between 2022 and 2023. During the six months prior to the study, a total of 14,734 patients visited these outpatient facilities. To ensure the reliability of the study, neither medical nor non-medical staff were informed about the planned data collection. Eighty-five samples were collected from frequently touched surfaces used by both patients and medical staff. These samples were divided into two categories: patient areas (53 samples) and staff areas (32 samples). The patient areas included surfaces such as the armrest of a blood collection chair, a tourniquet, the arm of a hand sanitizer dispenser for patients, the cuff of a blood pressure monitor, an infant scale, and the surface of a procedure table. The staff areas included surfaces such as a suction device handle, a blood collection table surface, a surgical lamp handle, the interior surface of a room containing sterile packs, and an auxiliary procedure table. Samples were collected from rooms categorized by the type of medical service provided. Procedure rooms included those where skin-breaking procedures were performed, such as blood collection and minor surgical procedures (e.g., mole removal, wound suturing, stitch removal, and wound dressing changes). Diagnostic rooms were defined as those used for non-invasive medical services like medical consultations, physical examinations, obstetric assessments, and radiological diagnostics. Other rooms included non-medical spaces such as mother-and-child rooms, hallways, and reception areas. A detailed list of sites of swab collection is provided in the Supplementary Materials.

Additionally, the study distinguished between medical procedure areas (e.g., blood collection, electrocardiogram procedures, wound dressing changes) comprising 45 samples, and non-medical areas (e.g., mother-and-child rooms, hallways, and reception areas) comprising 40 samples. Detailed of samples taken by variable are presented in Table 1.

In the outpatient facilities, cleaning services were performed by external cleaning companies, whereas disinfection was carried out by medical staff. In all studied facilities, cleaning procedures (including cleaning and disinfection frequency and methods) were conducted according to a standardized hygiene plan using the same cleaning and disinfecting agents. The medical personnel used ready-to-use alcohol-based and alcohol-free disinfectant solutions, as well as alcohol-based and alcohol-free disinfectant wipes. The ready-to-use solutions were applied using disposable paper towels. In contrast, the cleaning team used ready-to-use alcohol-based disinfectant solutions and diluted disinfectant solutions, which were applied using reusable cloths subjected to a laundering process. For surface disinfection, in addition to alcohol-based wipes, alcohol-free wipes containing hydrogen peroxide and didecyldimethylammonium chloride were used.

Samples containing only bacterial microbiota were classified as microbiota samples, while those with mixed strains (including microbiota) were classified as other.

### 2.3. Microbiological Analysis

To assess microbiological contamination of surfaces, swabs were taken from selected areas using a flocked e-Swab (Copan Italia SpA via Perotti 10, Brescia, Italy) moistened with sterile saline. Swabs from larger surfaces were taken from areas of approximately 25 cm^2^, while swabs from smaller surfaces, such as light switches, were taken from the entire element. The swab was placed in Amies liquid transport medium and delivered to the laboratory at a temperature of about 4 °C. The material was then plated onto Columbia blood agar (Becton, Dickinson and Company, Franklin Lakes, NJ, USA) and incubated for 24 h at 37 °C under aerobic conditions. After this time, the plates with bacterial growth were removed, colonies were counted, and colonies representing different species (visually distinguishable) were selected for species identification. Growth was evaluated quantitatively using three categories: no or scarce growth (up to 10 colonies), moderate growth (11 to 30 colonies), and abundant growth (more than 30 colonies). After plating onto solid media, the swabs were placed back into the transport medium and incubated under the same conditions as the plates for 24 h. If growth occurred the following day, bacteria were subcultured onto Columbia blood agar using the streak plate method, as with direct plating, and the results were evaluated after 24 h. In cases of bacterial growth in samples negative on direct plating, bacterial colonies were collected for identification. Identification was carried out using the MALDI-ToF mass spectroscopy method (Bruker Daltonik MALDI Biotyper, Billerica, MA, USA) in Korlab Medical Laboratories, Ruda Śląska, Poland.

A detailed description of the strains and their quantities categorized by growth is provided in the Supplementary Materials.

### 2.4. Ethical Considerations

The study was approved by the appropriate Research Ethics Committee of the Medical University of Warsaw, Poland (approval number: AKBE 226/2021, approval date: 13 December 2021). All participants, including medical staff and patients, were informed about the purpose of the study, and their data remained anonymous. Additionally, neither medical nor non-medical staff were aware of the exact timing of the planned sample collection, which minimized the risk of altering routine cleaning practices.

## 3. Results

Of the 85 samples collected, five showed no growth. The remaining samples were compared based on the presence of skin microbiota and spore-forming bacteria. The five cultures in which no growth occurred were excluded from the comparison.

The samples did not show statistically significant differences between each other in terms of city, disinfection, room, area, procedures involving breach of skin integrity, type of procedure, microbiological environmental cleanliness, or growth.

A statistically significant increase in moderate/heavy growth was observed in samples containing microbiota compared to those containing other microorganisms (n = 23, 52% vs. n = 7, 19.4%, *p* = 0.003). Similarly, there were more samples containing spore-forming bacteria compared to non-spore-forming bacteria (n = 17, 58.6% vs. n = 13, 23.2%, *p* = 0.001). A statistically significant difference was found between no or scarce growth and moderate or abundant growth in the microbiota and other groups (n = 21, 47.7% vs. n = 29, 80.6%, *p* = 0.003), as well as between no or scarce growth and moderate or abundant growth in the spore-forming and non-spore-forming groups (n = 12, 41.4% vs. n = 43, 76.8%, *p* = 0.001). Detailed results are presented in Table 2.

**Figure 1:**
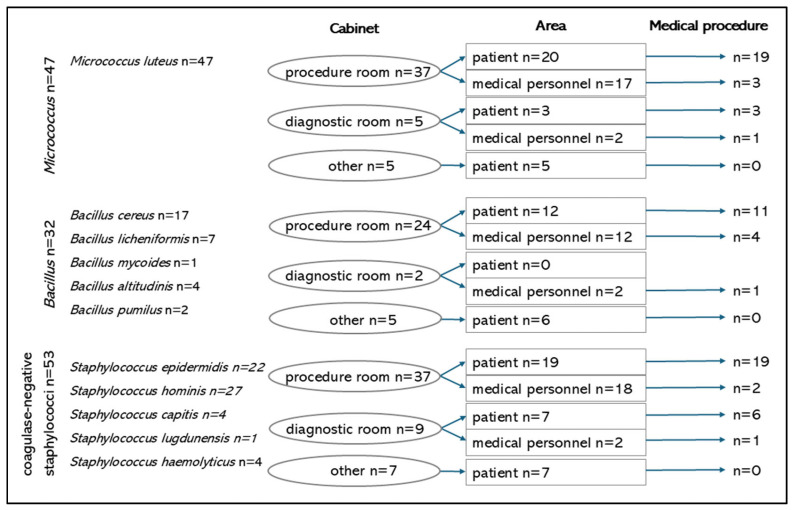
Distribution of bacterial genera and their prevalence in relation to the place with regard to designated areas and the performance of medical procedures.

Figure 1 presents a detailed distribution of bacterial genera and their prevalence in relation to the place (room) with regard to designated areas (patient/staff) and the performance of medical procedures. *Micrococcus luteus* was most frequently isolated in the procedure rooms (n = 37), predominantly within the patient zone (n = 20) where medical procedures were performed (n = 19). Similarly, coagulase-negative staphylococci (n = 53) were most commonly detected in the procedure room (n = 37), particularly in the patient zone (n = 19) in areas where medical procedures were conducted (n = 19).

An analysis of the number of visits made by patients at each center, categorized according to identified bacterial strains, was also performed. No statistically significant differences were found between the number of patient visits in the other/microbiota groups (Z = 0.684, *p* = 0.494), spore-forming/non-spore-forming groups (Z = −1.743, *p* = 0.080), and microbiological environmental cleanliness (Z = 0.124, *p* = 0.901).

## 4. Discussion

Hospital-acquired infections in settings such as outpatient clinics and hospitals represent a significant public health issue, impacting the patient’s quality of life and healthcare costs. As noted by the authors, this threat is not limited to hospitals but extends to outpatient care settings. An outpatient clinic is a place where, among other services, healthcare continues after a hospital stay. However, the duration of a patient’s stay in an outpatient facility is significantly shorter than a hospital stay, as it typically involves the provision of healthcare services that do not require 24-h or all-day treatment. Outpatient facilities also conduct procedures that breach tissue integrity, such as minor surgeries, including suture placement and removal, wound care and dressing changes, injections, and blood collection. Research has shown that microorganisms can be transmitted from medical staff to patients, from patients to medical staff, and from patient to patient, posing challenges in terms of infection control and disinfection [5]. However, most studies on HAIs focus on hospital environments, with studies on outpatient settings being rare [17]. This is due to the greater emphasis placed on hospitals, where the risk of infection transmission is well recognized, as opposed to outpatient facilities, where the risk is lower but still significant and requires monitoring. Therefore, our study is unique as it focuses on outpatient facilities rather than closed healthcare settings, as is the case in most studies. However, it should be noted that, just as in hospitals, direct contact transmission via the hands of healthcare personnel remains the primary mode of microorganism transmission [18]. In our study, most samples, as expected, showed scant growth with environmental and skin microbiota bacteria.

In an outpatient study by Reynolds, where microbiome transmission was traced using a harmless viral marker, it was found that after several hours, the greatest contamination occurred on the door handles of doctors’ offices and nurse station chairs [19]. Similarly, in our study, the most frequently touched surfaces were considered. We took into account not only areas directly related to healthcare services, such as procedure tables or tourniquets, but also light switches and refrigerator handles. Samples were also taken from office areas, such as the reception area and those dedicated for patient use (e.g., changing tables). In our samples, the highest number of microbiota and spore-forming strains was found in procedure rooms and samples unrelated to tissue integrity procedures. Spore-forming bacteria were more frequently found in the staff disinfection area in procedure rooms, as well as in procedures not related to breaking the continuity of the skin. A statistically significant difference was not found between the number of patient visits in the other/microbiota or spore-forming/non-spore-forming groups, or microbiological environmental cleanliness.

The bacterial flora found on the skin of healthcare workers are present in hospitals and office spaces. In a study by Li, skin microbiota and other bacteria were observed on office keyboards and mice [20]. However, cleaning and disinfection in healthcare facilities differ significantly from cleaning in public spaces such as hotels or offices [21]. In the hygiene plan used in the outpatient clinics studied, office surfaces (keyboards, mice, etc.) were cleaned using a cleaning/disinfecting agent and reusable wipes.

In our study, the growth of microbiota and spore-forming microorganisms, which accounted for one-third of all identified strains (29 strains), was observed. The most frequently identified strain was *Bacillus cereus*, which appeared in 19 samples (65.5%). Of these, 8 samples showed scant growth, while 11 exhibited moderate and heavy growth. Identified samples more often colonized areas cleaned by staff rather than by cleaning companies (60.1%, n = 18 vs. 37.9%, n = 11). This well-known pathogen (bacteria and/or their spores) is commonly found in the environment and food products, and its toxins can cause gastrointestinal illnesses, resulting in diarrhea and vomiting [22]. Veysseyre’s study analyzed 57 cases of patients hospitalized due to *Bacillus cereus* infections. HAIs were diagnosed in 38 patients (70.4%), suggesting that other potential sources of infection may exist outside the hospital [23].

Russotto’s study, conducted in an intensive care unit, indicated the presence of *Staphylococcus aureus* on stethoscopes, blood pressure cuffs, ultrasound machines, and ventilator switches [24]. In our study, *S. aureus* was found in one sample with high growth, located on a patient chair. In intensive care units, the bacterial load of *Staphylococcus* is significantly higher, partly due to the condition of the hospitalized patients and the severity of the infections.

Studies indicate that procedures, cleaning agents, and staff education are crucial for effective disinfection. Maintaining hygiene is a key factor in preventing infections, with disinfection of frequently touched surfaces playing a major role [19,21]. Research also emphasizes the need for a sound disinfection strategy considering risk profiles related to surfaces, patients, and pathogens, as well as addressing reduced susceptibility and increased bacterial resistance to disinfectants [25,26]. In all four outpatient facilities, standardized cleaning and disinfection procedures were implemented, specifying the scope and frequency of tasks. Cleaning, including disinfection, was conducted according to a set frequency and type of activity depending on the area, zone, and type of room using professional cleaning and disinfecting agents. The cleaning supplies used (e.g., wipes) were reusable, following standard hygiene protocols. Medical staff had unrestricted access to disinfectant wipes and rapid disinfection agents. It is also worth noting that most cleaning staff were migrants from Ukraine, which may have presented language barriers and difficulties in receiving appropriate training in hygiene procedures.

The pandemic has led to an increased demand for more effective infection prevention and control measures in all healthcare facilities [27]. Studies show an initial increase in hand hygiene compliance among healthcare workers during the early stages of the pandemic, followed by a subsequent decline [28]. In outpatient clinics, hand hygiene and other hygiene training during the pandemic was delivered online. This situation highlights the importance of ongoing hand hygiene training to maintain awareness of its significance. Based on the results of our study, hygiene procedures in medical centers have been revised. However, it should not be forgotten that proper hand hygiene, when practiced by medical staff and cleaning personnel, forms the foundation for all other hygiene procedures. Proper hand hygiene significantly reduces the transmission of microorganisms within healthcare settings.

## 5. Conclusions

The results of this study led to changes in cleaning procedures. Reusable wipes were replaced with disposable ones, and disinfectants were updated across all areas of the outpatient clinic. Additionally, the frequency of training for medical staff, including hand hygiene training, was increased. Concurrently, changes were made to the contracts with cleaning companies, specifying the frequency and scope of training and the language of materials adapted to the nationality of the cleaning staff. Detailed guidelines were developed for implementation of the hygiene plan regarding the use of disinfectants, handling of cleaning equipment, and execution of cleaning procedures, among other aspects. Systematic quality control of the cleaning company’s work was also introduced, and the scope of inspections was expanded to include the equipment used for cleaning, the compliance of disinfectants and cleaning agents with the Hygiene Plan, the proper preparation and application of these agents, and the inspection of the rooms used by the cleaning staff.

## Figures and Tables

**Table 1:** Summary of samples taken.

Category	Place	Number (%)
City	Cracow	53 (62.4%)
	Warsaw	32 (37.6%)
Disinfection	Cleaning company	23 (27.1%)
	Medical personnel	62 (72.9%)
Room	Procedure room	60 (70.6%)
	Diagnostic room	11 (12.9%)
	other	14 (16.5%)
Area	Patient	53 (62.4%)
	Medical personnel	32 (37.6%)
Procedures involving the breach of skin integrity	Yes	12 (14.1%)
	No	73 (85.9%)
Type of procedure	Medical	45 (52.9%)
	Non-medical	40 (47.1%)
Microbiological environmental cleanliness	None + scarce	55 (64.7%)
	Abundant	30 (35.3%)
Growth	None	5 (5.9%)
	Scarce	50 (58.8%)
	Moderate	13 (15.3%)
	Abundant	17 (20.0%)
Origin *	Procedure room	60 (70.6%)
	Diagnostic room	11 (12.9%)
	Other	14 (16.5%)

* potential presence of multiple strains in a single sample.

**Table 2:** Differences in the entire group based on the presence of microbiota vs. cultures with different species compositions.

Category	Place	Microbiota (n = 44)	Others (n = 36)	*p*	Spore- Forming (n = 29)	Non-Spore- Forming (n = 56)	*p*
City	Cracow	30 (68.2%)	18 (50%)	0.099	18 (62.1%)	35 (62.5%)	0.969
	Warsaw	14 (31.8%)	18 (50%)		11 (37.9%)	21 (37.5%)	
Disinfection	Cleaning company	31 (70.5%)	28 (77.8%)	0.459	11 (37.9%)	12 (21.4%)	0.104
	Medical personnel	13 (29.5%)	8 (22.2%)		18 (62.1%)	44 (78.6%)	
Room	Procedure room	33 (75%)	24 (69.7%)	0.409	21 (72.4%)	39 (69.6%)	0.419
	Diagnostic room	4 (9.1%)	7 (19.4%)		2 (6.9%)	9 (16.1%)	
	Other	7 (15.9%)	5 (13.9%)		6 (20.7%)	8 (14.3%)	
Area	Patient	28 (63.6%)	23 (63.9%)	0.981	17 (58.6%)	36 (64.3%)	0.610
	Medical personnel	16 (36.4%)	13 (36.1%)		12 (41.4%)	20 (35.7%)	
Procedures involving the breach of skin integrity	Yes	7 (15.9%)	3 (8.3%)	0.497	4 (13.8%)	8 (14.3%)	0.790
	No	37 (84.1%)	33 (91.7%)		25 (86.2%)	48 (85.7%)	
Type of procedure	Medical	25 (56.8%)	18 (50%)	0.702	14 (48.3%)	31 (55.4%)	0.535
	Non-medical	19 (43.2%)	18 (50%)		15 (51.7%)	25 (44.6%)	
Growth	No + scarce	21 (47.7%)	29 (80.6%)	0.003 *	12 (41.4%)	43 (76.8%)	0.001 *
	Moderate + abundant	23 (52.3%)	7 (19.4%)		17 (58.6%)	13 (23.2%)	

* *p* < 0.05.

## Data Availability

The original contributions presented in this study are included in the article/Supplementary Materials. Further inquiries can be directed to the corresponding authors.

## Supplementary Materials

The following supporting information can be downloaded at: https://www.mdpi.com/article/10.3390/microorganisms13030698/s1. Table S1. Detailed of sites of swab collection categorized by office groups and cleaning personnel; Table S2. Identified strains and their quantities categorized by growth.

## Abbreviations

HAIs I	Hospital-acquired infections
