# Supplementary material for: Microbial Contamination on High-Touch Surfaces in Outpatient Clinics: Identification of Bacterial Strains from Areas of Patient and Medical Staff Occupancy
Source: Microorganisms. 2025 Mar 20;13(3):698. doi: 10.3390/microorganisms13030698 (PMC11945162; doi:10.3390/microorganisms13030698)
Supplement: Supplementary file 1 [file microorganisms-13-00698-s001.zip › microorganisms-3450265-supplementary.pdf]

microorganisms

MDPI

Table S1. Detailed of sites of swab collection categorized by office groups and Cleaning Personnel.

Location/Responsible Personnel		Cleaning Personnel Assigned
treatment room	dispenser near sink (used by patient) (n=2)	cleaning company
	light fixture (n=2)	cleaning company
	refrigerator handle (n=4)	cleaning company
	blood pressure monitor (n=4)	medical personnel
	chair armrest for patient (n=3)	medical personnel
	ECG cables - other (n=2)	medical personnel
	ECG limb leads (n=4)	medical personnel
	keyboard (n=4)	medical personnel
	CTG cables (n=2)	medical personnel
	mouse (n=4)	medical personnel
	patient chair armrest (n=4)	medical personnel
	spirometer (n=2)	medical personnel
	suction device handle (n=3)	medical personnel
	suction device switch (n=2)	medical personnel
	tourniquet (n=4)	medical personnel
	surgical table (n=2)	medical personnel
	blood collection table (n=3)	medical personnel
	operating table (n=2)	medical personnel
	cabinet with sterile packages (n=2)	medical personnel
	infant scale (n=4)	medical personnel
diagnostic office	gynecological chair (n=2)	medical personnel
	ultrasound probe/cable (n=1)	medical personnel
	ultrasound machine cables (n=1)	medical personnel
	ultrasound probes (n=2)	medical personnel
	X-ray wall (n=2)	medical personnel
	X-ray wall handle (n=2)	medical personnel
	ultrasound probe (n=1)	medical personnel
	gynecological chair (n=2)	medical personnel
other	dispenser (n=3)	cleaning company
	patient chair (mother and child room) (n=1)	cleaning company
	changing table (n=4)	cleaning company
	self-check-in kiosk (n=4)	cleaning company
	light switch (n=2)	cleaning company

Table S2. Identified strains and their quantities categorized by growth.

Strains	None + Stingy (n=55)	Moderate + Abundant (n=30)
Moraxella catarrhalis	0	1 (3,33%)
Enterobacter cloacae	0	1 (3,33%)
Pantoea septica	0	1 (3,33%)
Rhizobium radiobacter	0	1 (3,33%)
Lysinibacillus fusiformis	0	1 (3,33%)
Paracoccus yeei	0	1 (3,33%)
Corynebacterium lipophiloflavum	0	1 (3,33%)
Erwinia sp	0	1 (3,33%)
Chryseobacterium gleum	0	1 (3,33%)

 https://doi.org/10.3390/microorganisms13030698

Roseomonas mucosa	0	1 (3,33%)
Pseudomonas luteola	0	1 (3,33%)
Pseudomonas stutzeri	0	1 (3,33%)
Bacillus mycoides	0	1 (3,33%)
Staphylococcus aureus	0	1 (3,33%)
Staphylococcus lugdunensis	0	1 (3,33%)
Bacillus pumilus	1 (1,82%)	1 (3,33%)
Staphylococcus haemolyticus	3 (5,45%)%)	1 (3,33%)
Bacillus altitudinis	2 (3,64%)	2 (6,67%)
Staphylococcus capitis	2 (3,64%)	2 (6,67%)
Kocuria rhizophila	1 (1,82%)	4 (13,33%)
Bacillus licheniformis	3 (5,45%)	4 (13,33%)
Staphylococcus warneri	10 (18,18%)	5 (16,67%)
Staphylococcus hominis	18 (32,73%)	9 (30,00%)
Bacillus cereus	8 (14,55%)	11 (36,67%)
Staphylococcus epidermidis	11 (20,00%)	11 (36,67%)
Micrococcus luteus	26 (47,27%)	21 (70,00%)
